# Editorial: Pediatric Obesity: A Focus on Treatment Options

**DOI:** 10.3389/fped.2019.00177

**Published:** 2019-05-07

**Authors:** Fatima Cody Stanford, Angela K. Fitch

**Affiliations:** ^1^MGH Weight Center, Massachusetts General Hospital, Boston, MA, United States; ^2^Neuroendocrine Unit, Division of Endocrinology, Departments of Medicine and Pediatrics, Boston, MA, United States; ^3^Harvard Medical School, Boston, MA, United States; ^4^Endocrine Unit, Division of Endocrinology, Department of Medicine, Boston, MA, United States

**Keywords:** obesity, pediatric obesity, nutrition, weight loss medications, weight loss surgery, bariatric surgery

Obesity is the most prevalent chronic disease in the United States with 17.8% of youth aged 2–19 with obesity and 5.8% with severe obesity (1). In 2015, it was noted that the average weight of children had risen an average of more than 5 kg within 3 decades, but it is also noteworthy that low-income and middle income countries have reported similar or even more rapid increases in the prevalence of obesity (2, 3). While it is important to continue efforts to promote prevention of obesity in children and adolescents, we must ensure that efforts also target the treatment of obesity to ensure that gains in life expectancy throughout the world do not decline due to the deleterious impacts of this chronic disease of obesity. Our issue seeks to address issues surrounding treatment of obesity in the pediatric population.

In our topic, there are three reviews on: (1) genetics and the role it plays in obesity, (2) vascular disease and atherosclerosis and its role in fatty liver in the pediatric population, and (3) the role of pediatric obesity in eating disorders. Genetics play a large role in the development of obesity, but its role in common (multifactorial/polygenic) obesity is less pronounced than in persons with monogenic and syndromic obesity (4). Mǎrginean and colleagues review genetic and obesogenic environmental factors and the roles they play in the prevalence of childhood obesity in their review, Mǎrginean et al. In Karjoo seeks to discern if there is a correlation between atherosclerosis and fatty liver disease in adolescents due to the early incidence of these entities in children and adolescents. Finally, De Giuseppe and colleagues evaluate the impact of multi-disciplinary treatment of eating disorders to decrease obesity in this population in De Giuseppe et al.

With the rise in obesity in the pediatric population, are patients being adequately referred for management of their obesity? Imoisili and colleagues seek to provide insights about childhood obesity referral types as it relates to clinicians, clinical practice, and patient characteristics in Imoisili et al. When pediatric patients with overweight and obesity are evaluated, is there a clear algorithm that clinicians may follow to ensure standardized care? Cuda and Censani present a pediatric algorithm for the care of patients with obesity in Cuda and Censani.

In Schumaker and Censani present the case of a 10 year old male with severe obesity who presented with growth failure and excessive weight gain and treatment strategies for the care of this patient. Outside of behavioral and lifestyle therapies, there is often minimal use of evidence-based treatments for pediatric patients with obesity in the US and around the world. In Fox and Kelly demonstrate the utility of weight loss medications in the treatment of obesity in the pediatric population. Dr. Campoverde Reyes and colleagues evaluate weight loss surgery utilization in adolescents and young adults in several academic institutions in Campoverde Reyes et al. to ascertain whether the most effective treatment for moderate to severe obesity, metabolic and bariatric surgery, is utilized in the patients most likely to garner benefit. When metabolic and bariatric surgery is utilized in adolescents and young adults, it is important to recognize that there is often an overestimation of resting energy expenditure (REE), a tool that may be utilized to govern behavioral strategies in the postoperative setting, as noted by Rickard et al.

It is well-known that racial and ethnic minority populations have disproportionately higher levels of obesity and subsequent lower levels of treatment compared to majority populations (5). As such, Srivastava and colleagues evaluated the feasibility of shared medical appointments (SMA) in African-American families with obesity in an urban safety net hospital in their original research, Srivastava et al. Thornton and colleagues also evaluated chronic disease management for diseases such as obesity in families and how it plays a role in adolescent family members and their health behaviors in Thornton et al. Finally, Showell and colleagues sought to examine the association between neighborhood factors and obesity in overweight in racial and ethnic minority preschool children in Showell et al.

With this issue, Stanford and Fitch we have presented a wide spectrum of work surrounding the diagnosis and treatment of obesity in diverse pediatric populations. Much is needed to ensure that children, adolescents, and young adults receive adequate care for overweight or obesity.

## Author Contributions

FS drafted and reviewed the manuscript. AF reviewed the manuscript.

### Conflict of Interest Statement

The authors declare that the research was conducted in the absence of any commercial or financial relationships that could be construed as a potential conflict of interest. The reviewer HC declared a past co-authorship with one of the authors, FS, to the handling Editor.
